# Impact of Drug Stock-Outs on Death and Retention to Care among HIV-Infected Patients on Combination Antiretroviral Therapy in Abidjan, Côte d'Ivoire

**DOI:** 10.1371/journal.pone.0013414

**Published:** 2010-10-15

**Authors:** Armelle Pasquet, Eugène Messou, Delphine Gabillard, Albert Minga, Ayeby Depoulosky, Sylvie Deuffic-Burban, Elena Losina, Kenneth A. Freedberg, Christine Danel, Xavier Anglaret, Yazdan Yazdanpanah

**Affiliations:** 1 Service Universitaire des Maladies Infectieuses et du Voyageur, Centre Hospitalier de Tourcoing, Tourcoing, France; 2 Programme Aconda, Abidjan, Côte d'Ivoire; 3 INSERM U897, Bordeaux, France; 4 Programme PACCI, Abidjan, Côte d'Ivoire; 5 INSERM U995, Lille, France; 6 EA 2694, Faculté de Médecine de Lille, France; 7 Massachusetts General Hospital, Boston, Massachusetts, United States of America; University of Cape Town, South Africa

## Abstract

**Background:**

To evaluate the type and frequency of antiretroviral drug stock-outs, and their impact on death and interruption in care among HIV-infected patients in Abidjan, Côte d'Ivoire.

**Methods and Findings:**

We conducted a cohort study of patients who initiated combination antiretroviral therapy (cART) in three adult HIV clinics between February 1, 2006 and June 1, 2007. Follow-up ended on February 1, 2008. The primary outcome was cART regimen modification, defined as at least one drug substitution, or discontinuation for at least one month due to drug stock-outs at the clinic pharmacy. The secondary outcome for patients who were on cART for at least six months was interruption in care, or death. A Cox regression model with time-dependent variables was used to assess the impact of antiretroviral drug stock-outs on interruption in care or death. Overall, 1,554 adults initiated cART and were followed for a mean of 13.2 months. During this time, 72 patients discontinued treatment and 98 modified their regimen because of drug stock-outs. Stock-outs involved nevirapine and fixed-dose combination zidovudine/lamivudine in 27% and 51% of cases. Of 1,554 patients, 839 (54%) initiated cART with fixed-dose stavudine/lamivudine/nevirapine and did not face stock-outs during the study period. Among the 975 patients who were on cART for at least six months, stock-out-related cART discontinuations increased the risk of interruption in care or death (adjusted hazard ratio [HR], 2.83; 95%CI, 1.25–6.44) but cART modifications did not (adjusted HR, 1.21; 95%CI, 0.46–3.16).

**Conclusions:**

cART stock-outs affected at least 11% of population on treatment. Treatment discontinuations due to stock-outs were frequent and doubled the risk of interruption in care or death. These stock-outs did not involve the most common first-line regimen. As access to cART continues to increase in sub-Saharan Africa, first-line regimens should be standardized to decrease the probability of drug stock-outs.

## Introduction

The World Health Organization (WHO) and the Joint United Nations Program on HIV/AIDS (UNAIDS) estimate that in late 2007, 30–36 million people were living with HIV, including 22 million in sub-Saharan Africa and 480,000 in Côte d'Ivoire [1]. The dramatic rise in global funding for HIV/AIDS, as well as reduced drug costs, have increased the availability of combination antiretroviral therapy (cART) in sub-Saharan Africa [2], [3]. The number of HIV-infected patients on cART in this region has increased sharply from 100,000 in 2002 to 3 million in 2007, of whom 50,000 are in Côte d'Ivoire [4].

With increased access to cART, drug stock-outs related to insufficient human resources and poor infrastructure have been reported, leading to treatment modifications or discontinuations [5], [6], [7]. In an analysis of the determinants of adherence to cART among HIV-infected patients in Côte d'Ivoire, Diabaté *et al.* reported that 10% of interruptions were related to drug stock-outs [7]. The efficacy of cART relies on excellent adherence [8], since cART interruptions are associated with higher rates of drug resistance [9], opportunistic infections, and death [10].

Studies on the frequency and impact of cART stock-outs in sub-Saharan Africa are scarce. The objectives of this study are to assess the frequency of cART stock-outs in three major HIV care centers in Abidjan, Côte d'Ivoire; to determine whether stock-outs led to cART modifications or discontinuations; and to study the impact of stock-out-related modifications or discontinuations on retention in care and mortality.

## Methods

### Population

**Figure 1:**
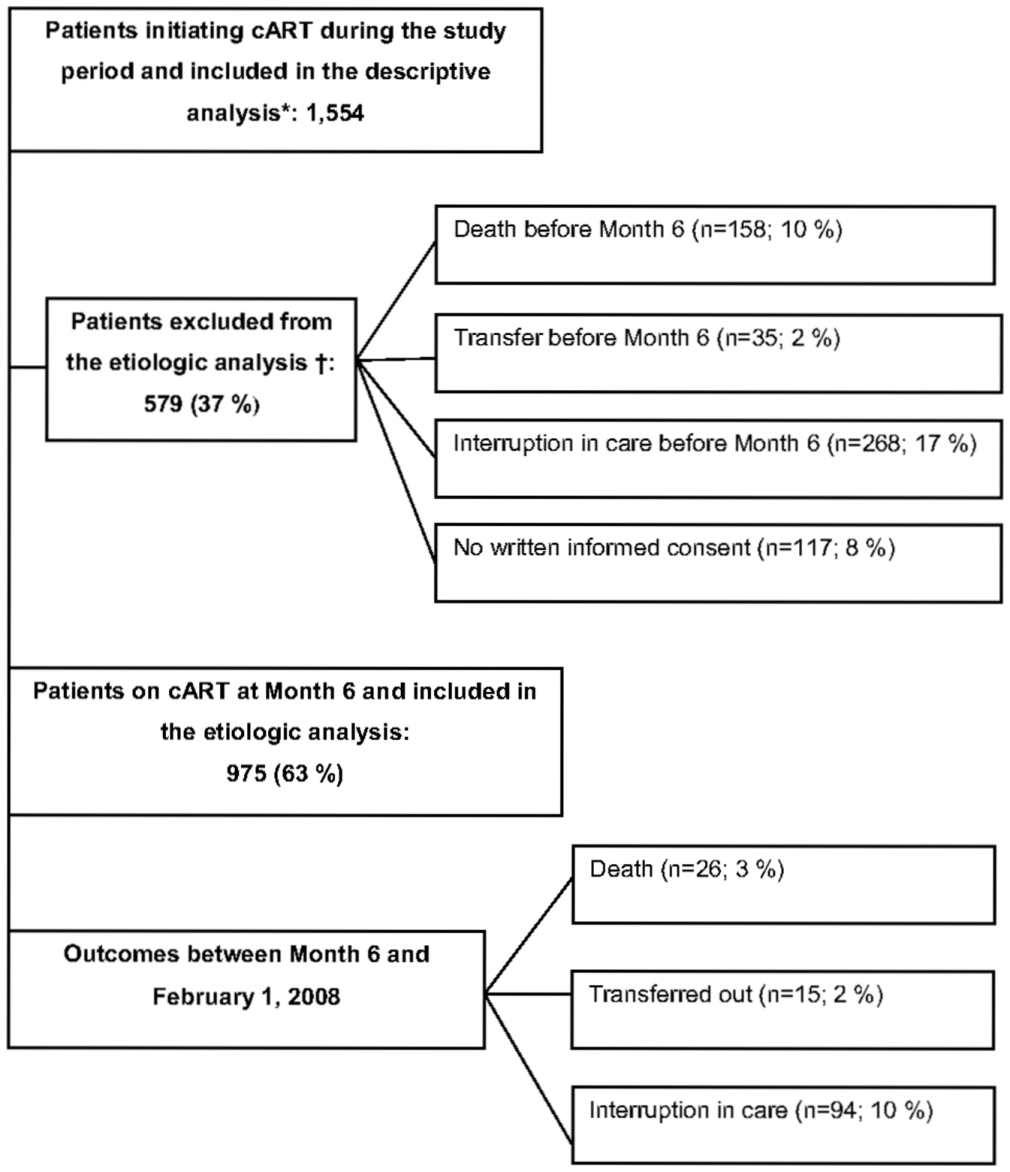
HIV-infected patients on cART in three Abidjan' HIV care centers, February 2006–February 2008. * The descriptive analysis evaluates the frequency of treatment modifications and discontinuation that are related to drug stock-outs. † The etiologic analysis evaluates the association between stock-out-related regimen modification or discontinuation and interruption in care or death. cART: combination antiretroviral therapy.

In 2004, Aconda, an Abidjan-based non-governmental organization, initiated a program of access to care and treatment aimed at HIV-infected patients [11], [12]. We conducted a prospective cohort study at three Aconda HIV care centers in Abidjan, Côte d'Ivoire: the Yopougon-Attié clinic, the CePReF clinic [12] and the HIV clinic affiliated with the National Center for Blood Transfusion (CNTS) [13]. Yopougon-Attié is a general medicine outpatient clinic, while CePReF and CNTS are outpatient clinics entirely devoted to HIV care. Study subjects were HIV-1 or HIV-1+2-infected adults aged ≥21 years (i.e; legal age in Côte d'Ivoire) who initiated cART between February 1, 2006 and June 1, 2007 in any of the three study centers and who provided written informed consent (**Figure 1**). We assessed the incidence and determinants of virologic resistance to cART in these patients. Follow-up began at cART initiation and ended when the database closed, on February 1, 2008.

### Ethics statement

The Aconda computerized data management system has been previously described [11], [12], and has been approved by the National Ethics Committee of Côte d'Ivoire. This study was conducted according to the principles expressed in the Declaration of Helsinki. All patients provided written informed consent for the collection of samples and subsequent analysis.

### Definitions

#### Drug stock-out

Patients were retrospectively considered to be affected by a drug stock-out if: (i) it led to a cART regimen modification, defined as at least one drug substitution, or an discontinuation of at least one month (i.e = prolonged discontinuation); (ii) the medical record stated that the cART modification or discontinuation had occurred due to a drug stock-out; and (iii) the cART modification or prolonged discontinuation occurred when there was a shortage of one of the patient's drugs, as reported by the study center pharmacy. We identified drug shortage periods by analyzing pharmacy data sheets.

#### Interruption in care

Patients were considered to have an interruption in care if they: (i) did not attend scheduled clinic visits for ≥3 months; (ii) did not return to care before the study ended on February 1, 2008; (iii) were not known to be in care at any other hospital or clinic during the study period; and (iv) were not known to have died [12], [14]. When patients did not show up for a scheduled visit, social workers and organizations of persons living with HIV/AIDS (PLWHA) tried to locate them by telephone or home visits [15].

### Data collection

We used a standardized monitoring system to collect detailed demographic, clinical, laboratory and therapeutic information on subjects in each study center. Data collected at enrollment included age, sex, marital status, education level, phone number and address, WHO clinical stage, CD4 count, and first-line cART regimen. Physicians and trained technicians collected follow-up data at each monthly visit. These data included AIDS-defining events, cART modification or discontinuation, and cause of modification or discontinuation. HIV RNA tests and CD4 counts were performed every six months and data were monitored with a standardized system.

### Statistical analyses

First, we described the baseline characteristics of patients enrolled in the study. We described quantitative variables using medians and interquartile ranges (IQR), and compared results from each study center using a nonparametric Wilcoxon rank-sum test. We summarized qualitative variables using percentages and compared results from each center using a Chi square test.

Second, we analyzed the association between stock-out-related treatment modification or prolonged discontinuation and interruption in care or death in the 975 patients who were on cART for at least six months during the study period (**Figure 1**). We restricted the analysis to this period for two reasons. First, most stock-outs in Aconda centers occurred between April 1, 2007 and October 15, 2007, when most patients had been on cART for at least six months. Second, mortality among patients on cART in sub-Saharan Africa is consistently higher during the first six months of treatment, when it is more likely to be related to other factors, including treatment initiation at low CD4 counts [12], [14], [15], [16], [17], [18], [19], [20]. The factors associated with treatment outcomes are thus different in the first six months and the last six months of treatment, and our database was more appropriate for analyzing the later period.

We did not separate retention in care and death into separate outcomes for two reasons. First, a large proportion of patients who were lost to follow-up likely represent unrecorded deaths as has been reported elsewhere[15], [18]. Second, we evaluated the impact of drug stock-outs on retention in care and death in a subset of patients who were on cART for at least six months. If we considered these two outcomes separately, the power of the analysis would be greatly reduced.

The interruption in care rate was defined as the number of patients who interrupted care, divided by the total number of person-years at risk of interruption in care. Patients were considered to be at risk of interruption in care from six months after cART initiation to the date of their last visit, transfer to a different HIV care center, interruption in care, or death. Patients who died were censored on the date of death, patients who transferred to a different HIV care center were censored on the date of their last visit at the study center, and patients who were alive and in care were censored on February 1, 2008. We estimated 95% confidence intervals using Poisson's exact distribution [21].

We stratified time to interruption in care or death according to patient characteristics. We used the Kaplan-Meier method to perform a time-to-event analysis, and a log-rank test to compare survival distributions.

We used a multivariate Cox proportional hazard regression model to examine the association between interruption in care or death and stock-out-related cART modification or discontinuation. We accounted for variations in exposure over time by making the “modification or discontinuation” variable time-dependent in the multivariate model [22]. The variable could have one of five values: (i) no cART discontinuation or modification (reference group); (ii) cART regimen modification due to stock-out; (iii) cART regimen modification not due to stock-out; (iv) cART discontinuation due to stock-out; and (v) cART discontinuation not due to stock-out. Patients who discontinued and modified their cART regimens were classified as having discontinued cART, even if the modification occurred before the discontinuation.

We also examined the association between interruption in care or death and sociodemographic variables at enrolment, as well as clinical/biological variables six months after cART initiation, using a multivariate Cox proportional hazard regression model. Quantitative variables were added to the model by category, to reduce the impact of the assumptions involved in log-linear modelling. The categories included in the multivariate model included age (above or below the median), body mass index (in quartiles) and CD4 count (in clinical relevant strata). A missing values category was created for the following variables: marital status, distance from study center, education level, and WHO clinical stage.

We added variables that were found to be associated with interruption in care or death in univariate analysis (p-value <0.25), to the multivariate model, in order to evaluate multiple factors simultaneously. We developed the multivariate model using a backward stepwise regression analysis [23]. In a multivariate analysis that used Wald's test model, results were considered to be significant when p-values were <0.05. In this analysis, to assess proportionality we first generate time dependant covariates by creating interactions of the predictors and a function of survival. We included the resulting time dependent covariates into the Cox model. Analyses were conducted with SAS® software, version 9.1 (SAS institute Inc. Cary, North Carolina, USA

### Sensitivity analyses

We conducted a sensitivity analysis on the definition of drug stock-out, because the reason for cART modification or discontinuation was not always noted in the medical record. In this sensitivity analysis, drug stock-out was defined as any treatment prolonged discontinuation (more than one month), was not accompanied by a specific explanation in the medical record, and occurred during a period of shortages in one of the patient's antiretroviral drugs, as reported by the study center pharmacy. In a second sensitivity analysis, we only included patients on cART regimens that included drugs affected by drug stock-outs, because drug stock-outs did not occur for all cART combinations.

## Results

Overall, 1,554 patients initiated cART between February 1, 2006 and June 1, 2007. Baseline characteristics are shown in **Table 1**. Median age was 36 years (IQR, 26–43), 73% of patients were female, and 92% of patients lived in Abidjan. At treatment initiation, 210 patients (15%) were at WHO clinical stage 4 and median CD4 count was 136/ml (IQR, 57–220). Fifty four percent of patients initiated cART with generic fixed-dose combination stavudine/lamivudine/nevirapine. Patient characteristics differed by study center. For example, patients at Yopougon-Attié started cART with more advanced disease than in other centers (WHO clinical stage 4: Yopougon-Attié, 29% compared to CePReF, 9% and CNTS, 9%; p<0.01). Patients at Yopougon-Attié also received stavudine/lamivudine/nevirapine less often than patients in the other centers (Yopougon-Attié, 20% compared to CePReF, 67% and CNTS, 83%; p<0.01). Patients were followed for a median of 13 months after starting cART (IQR, 7–18). Among these 1,554 patients, during the study period, 184 patients died (12%) and 362 had interrupted care (23%).

**Table 1:** Baseline characteristics of 1,554 HIV-infected adults who started cART in HIV care centers of the Aconda program in Abidjan, Côte d'Ivoire.

	CePReF	Yopougon-Attie	CNTS	Total ‡	p
**Mean, years/**mean (IQR)/N = 1553‡	36.1 (30.2–42.9)	35.3 (29.5–42.3)	35.2 (30,3–42,6)	35(25.9–42.7)	0.57^†^
**Gender, n (%)**	**904**	**485**	**162**	**1551**	**<0.01** *
Female	651(72.0)	379 (78.1)	109 (67.3)	1139 (73.4)	
**Marital status, n (%)**	**899**	**362**	**160**	**1421**	**<0.01**
Single	356 (39.6)	224 (61.9)	84 (52.5)	664 (46.7)	
Married	501 (55.7)	76 (21.0)	57 (35.6)	634 (44.6)	
Divorced or widower	42 (4.7)	62 (17.1)	19 (11.9)	123 (8.7)	
**Educational level, n (%)**	**631**	**303**	**116**	**1050**	0.17
Illiterate or primary school	238 (37.7)	133 (43.9)	35 (30.2)	406 (38.7)	
Secondary school or higher	393 (62.3)	170 (56.1)	81 (69.8)	644 (61.3)	
**Distance to care center, n (%)**	**860**	**467**	**155**	**1482**	**<0.01**
<50 km, from Abidjan	777 (90.3)	451 (96.6)	140 (90.3)	1368 (92.3)	
50–250 km	41 (4.8)	9 (1.9)	10 (6.5)	60 (4.0)	
>250 km	42 (4.9)	7 (1.5)	5 (3.2)	54 (3.7)	
**WHO clinical stage, n (%)**	**870**	**432**	**90**	**1392**	**<0.01**
1	29 (3.3)	2 (0.5)	31 (34.4)	62 (4.4)	
2	132 (15.2)	53 (12.3)	33 (36.7)	218 (15.7)	
3	632 (72.6)	252 (58.3)	18 (20.0)	902 (64.8)	
4	77 (8.9)	125 (28.9)	8 (8.9)	210 (15.1)	
**BMI/** mean (IQR)/ N = 1064‡	19.8 (17.8–22.2)	19.9 (17.6–22.4)	21.0 (18.4–23.1)	19.9 (17.8–22.4)	0.04
**CD4** cells/ml/**mean** (IQR)/N = 1520‡	139 (54–222)	135 (59–233)	132 (60–184)	136 (57–220)	0.14
**First-line ART regimen, n (%)**	**903**	**487**	**162**	**1552**	**<0.01**
ZDV/3TC/EFV	176 (19.4)	233 (47.9)	9 (5.6)	418 (26.9)	
d4T/3TC/EFV	74 (8.2)	78 (16.0)	11 (6.8)	163 (10.5)	
d4T/3TC/NVP	605 (67.0)	100 (20.5)	134 (82.7)	839 (54.1)	
ZDV/3TC/NVP	5 (0.6)	33 (6.8)	0	38 (2.4)	
Other	43 (4.8)	43 (8.8)	8 (4.9)	94 (6.1)	

*χ^2^ test; † Wilcoxon rank-sum test.

‡ Numbers do not add up to 1,554 because of missing values.

CNTS: HIV clinic affiliated with the National Center for Blood Transfusion;

ZDV: zidovudine; 3TC: lamivudine; d4T: stavudine; EFV: efavirenz; NVP: nevirapine.

Other cART regimens: zidovudine-lamivudine-indinavir/ritonavir, lamivudine-stavudine-indinavir/ritonavir, or stavudine-abacavir-efavirenz.

BMI: Body Mass Index; virological failure: plasma HIV RNA>300 copies/ml.

IQR: Inter quartile range.

Of the 1,554 study subjects, 830 (53%) discontinued cART for more than one month and 280 (18%) modified their cART regimens at least once. Drug stock-outs led to 72 prolonged treatment discontinuations and 98 regimen modifications, representing 11% of the 1,554 patients who started cART between February 1, 2006 and February 1, 2007. These stock-outs were responsible for 9% of all cART discontinuations and 30% of all cART regimen modifications. Median time from cART initiation to first prolonged stock-out-related discontinuation or modification was 9 months (IQR, 5–13). Twelve percent of prolonged discontinuations were not related to drug stock-outs, but rather to travel, funerals, adverse events, treatment with traditional remedies, or inability to pay for drugs. The reason for treatment discontinuation was not noted in the medical records of 657 patients (79%). For 53 of these patients, cART discontinuations occurred when at least one of the drugs in their regimen was not in stock at the study center pharmacy (**Table 2**). When we assumed that these patients interrupted cART because of drug stock-outs, an estimated 16% of cART discontinuations were due to drug stock-outs. Most cART regimen modifications at CePReF and CNTS occurred because of cART-related adverse events (48%). At Yopougon-Attié, the majority of modifications occurred because of drug stock-outs (54% compared to 26% at CePReF and 19% at CNTS; p<0.01).

**Table 2:** Reasons for treatment modifications and discontinuations, by period, in 1,554 HIV-infected adults who started cART in HIV care centers of the Aconda program in Abidjan, Côte d'Ivoire.

	Before 6 months n (%)	After 6 months n (%)	Total n (%)
**Reasons for cART modification**	N = 52	N = 277	N = 329*
Adverse event	24 (46.1)	133 (48.0)	157 (47.4)
Drug stock-out	10 (19.2)	88 (31.8)	98 (30.0)
Tuberculosis	14 (26.9)	22 (7.9)	36 (11.0)
Pregnancy	0 (0)	13 (4.7)	13 (4.0)
Treatment failure	0 (0)	6 (2.2)	6 (1.8)
Unknown	4 (7.7)	15 (5.4)	19 (5.8)
**Reasons for prolonged cART discontinuations**	N = 295	N = 535	N = 830
Drug stock-out	8 (2.7)	65 (12.2)	73 (8.8)
Other†	42 (14.2)	58 (10.8)	100 (12.1)
Unknown	245 (83.1)	412 (77.0)	657 (79.1)
*Not during antiretroviral drug stock-out period*	*229 (77.6)*	*375 (70.1)*	*604 (72.8)*
*During antiretroviral drug stock-out period*	*16 (5.4)*	*37 (6.9)*	*53 (6.3)*

*: The total number of patients who modified their treatment is 280. Total of cART modifications, different from 280 because patients can undergo several modifications.

† : Travel, adverse events occurrence, funeral, treatment with traditional remedies, inability to pay for drugs.

**Figure 2:**
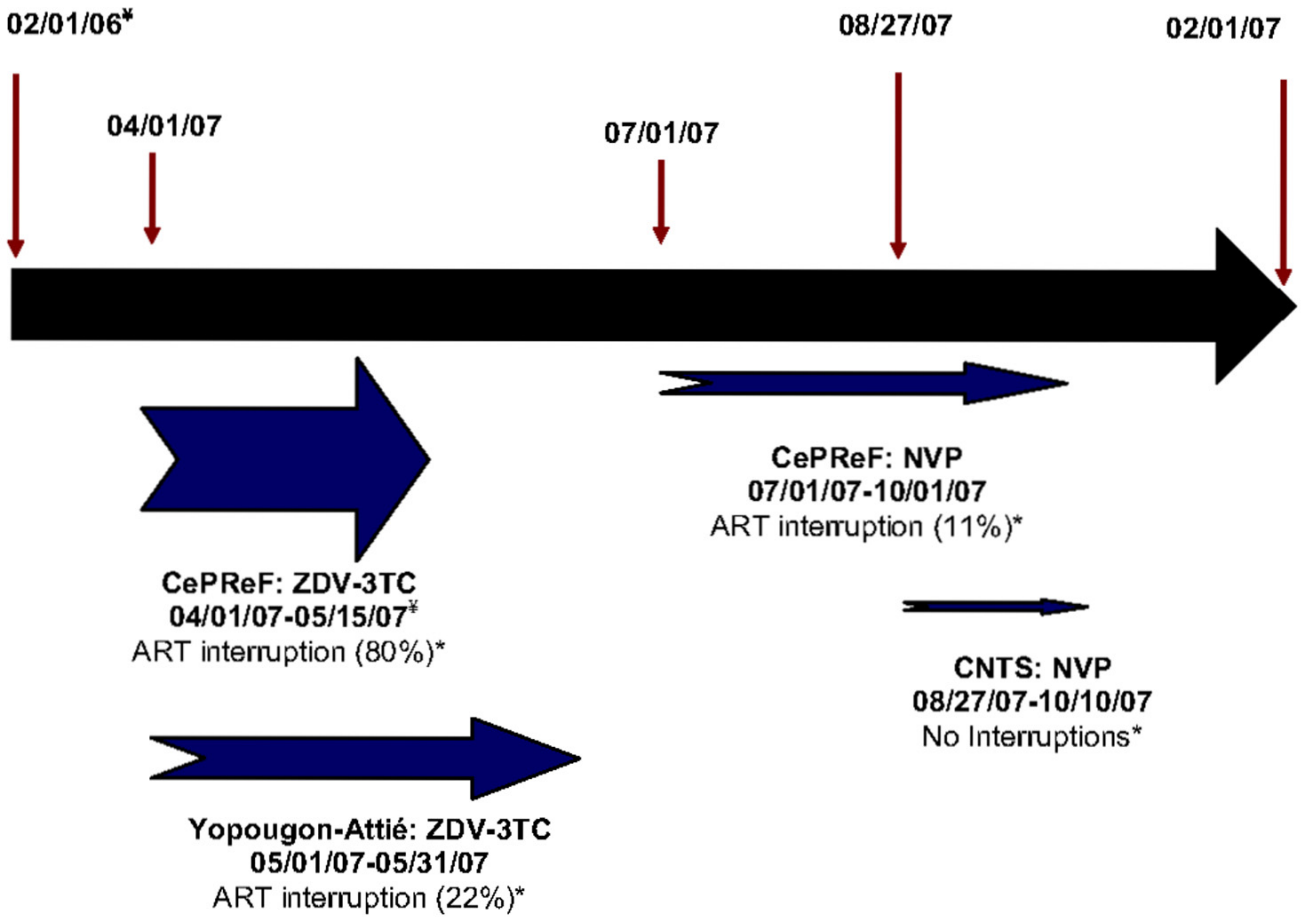
Diagram of main drugs affected by stock-outs in the Aconda program, February 2006–February 2008. CNTS: HIV care center affiliated with the National Center for Blood Transfusion. NVP: Nevirapine. ZDV-3TC: Zidovudine/lamivudine. * Proportion of patients who interrupt antiretroviral therapy = number of patients who interrupt therapy due to drug stock-outs/number on that therapy during this period. ¥ Month/day/year.

The first major drug stock-out was a shortage of fixed-dose zidovudine/lamivudine from April 1 to May 15, 2007 at CePReF and from May 1 to May 31, 2007 at Yopougon-Attié (**Figure 2**). There was also a shortage of nevirapine from July 1 to September 15, 2007 at CePReF and from August 27 to October 10, 2007 at CNTS. Patients taking nevirapine and fixed-dose zidovudine/lamivudine were affected by stock-outs most frequently, in 27% and 51% of cases, respectively. Stavudine/lamivudine/nevirapine, the most frequently prescribed regimen was not associated with any stock-outs and was always available. The proportion of stock-out-related cART discontinuations decreased over time (**Figure 2**; Cochran-Armitage test for trend p<0.01).

### Outcomes six months after cART initiation

Overall, 975 patients were followed for at least six months (**Figure 1**). Median CD4 count six months after cART initiation was 295/ml (IQR, 196–432) and 20% of patients had plasma HIV RNA>300 copies/ml. Compared to patients who died, had interrupted care, or transferred to a different HIV care center within the first six months of cART initiation , patients who remained on cART were older at cART initiation (37 versus 35 years; p = 0.03), had less advanced HIV disease (WHO Stage 4, 12% *vs.* 22%; p<0.01), and were more likely to have initiated cART with fixed-dose stavudine/lamivudine/nevirapine (58% *vs.* 46%; p<0.01).

Patients were followed for a mean additional time of 10 months (IQR: 6–13 months) after being followed on cART for six months. During this time, 26 patients died and 94 had interruption in care (**Figure 1**). The incidence of interruption in care was estimated at 14.5 per 100 person-years (PY; 95% CI, 14.2–14.7) and the mortality rate was 3.5/100PY (95% CI, 2.8–4.2). In patients who were still on cART six months after initiation, the probability of being alive and in care 18 months after cART initiation was 0.85 (95% CI, 0.82–0.87).

Of the 975 patients who were on cART for at least six months, 148 (15%) discontinued or modified their regimen because of drug stock-outs. As shown in **Table 3**, stock-out-related discontinuations were independently associated with a higher risk of interruption in care or death compared to no discontinuations or modifications (adjusted hazard ratio [HR], 2.83; 95% CI, 1.25–6.44). In contrast, stock-out-related regimen modifications did not have a significant impact on the outcome (adjusted HR, 1.21; 95% CI, 0.46–3.16). Other factors associated with an increased risk of interruption in care or death were plasma HIV RNA>300 copies/ml at month 6 (adjusted HR, 2.24; 95% CI, 1.50–3.33), cART regimens that did not contain any non-nucleoside reverse transcriptase inhibitors (NNRTI; adjusted HR, 2.49; 95% CI, 1.31–4.71), cART regimen modifications not related to drug stock-outs (adjusted HR, 3.38; 95% CI, 1.47–7.44) and cART discontinuations not related to drug stock-outs (adjusted HR, 2.78; 95% CI, 1.77–4.37). Interruption of care and death were not significantly associated with study center, age, gender, distance from HIV care center, WHO clinical stage or baseline CD4 counts.

**Table 3:** Factors independently associated with interruption in care or death among 975 HIV-infected patients in care 6 months after cART initiation, Abidjan, Côte d'Ivoire; basecase and sensitivity analysis on drug stock-out definition.

	Basecase Analysis					Sensitivity analysis**		
	Interruption in care/death, n (%)		HR	95% CI	p	HR	95% CI	p
	Yes	No						
**cART discontinuation or modification**								
None	30 (8.82)	310 (91.18)	1.00		**<0.01** *	1.00		**<0.01** *
Modification/stock-out	5 (5.95)	79 (94.05)	1.21	0.46–3.16	0.70	1.22	0.46–3.02	0.68
Modification/other reasons	7 (11.29)	55 (88.71)	3.38	1.47–7.74	**<0.01**	3.40	1.49–7.80	**0.01**
Discontinuation/stock-out	8 (12.50)	56 (87.50)	2.83	1.25–6.44	**0.01**	3.09	1.36–7.05	**0.01**
Discontinuation/other reasons	69 (16.39)	352 (83.61)	2.78	1.77–4.37	**<0.01**	2.80	1.78–4.40	**<0.01**
**Study center**								
CePReF	56 (8.90)	573 (91.10)	1.00		0.13*	1.00		0.13*
CNTS	17 (17.17)	82 (82.83)	1.52	0.86–2.69	0.15	1.52	0.86–2.69	0.15
Yopougon-Attie	46 (18.93)	197 (81.07)	1.53	0.95–2.45	0.08	1.54	0.96–2.47	0.07
**First-line cART regimen**								
d4T/3TC/NVP	59 (10.39)	509 (89.61)	1.00		**0.02** *	1.00		**0.02** *
ZDV/3TC/EFV	29 (11.65)	220 (88.35)	0.93	0.56–1.56	0.78	0.91	0.55–1.53	0.73
d4T/3TC/EFV	14 (15.05)	79 (84.95)	1.71	0.92–3.17	0.09	1.71	0.92–3.17	0.09
ZDV/3TC/NVP	4 (23.53)	13 (76.47)	1.44	0.49–4.25	0.51	1.41	0.48–4.18	0.53
Other	13 (29.55)	31 (70.45)	2.49	1.31–4.71	**<0.01**	2.46	1.30–4.67	**0.01**
**Virological failure (6 months)**								
No	78 (9.87)	712 (90.13)	1.00		**<0.01**	1.00		**<0.01**
Yes	41 (22.65)	140 (77.35)	2.24	1.50–3.33		2.24	1.50–3.33	

HR: hazard ratio, adjusted for all variables in the table;

CNTS: HIV clinic affiliated with the National Center for Blood Transfusion;

ZDV: zidovudine; 3TC: lamivudine; d4T: stavudine; EFV: efavirenz; NVP: nevirapine.

Other cART regimens: zidovudine-lamivudine-indinavir/ritonavir, lamivudine-stavudine-indinavir/ritonavir, or stavudine-abacavir-efavirenz.

Other reasons for modifications are: adverse events, tuberculosis, pregnancy and treatment failure. The reason for modification was unknown for 18 patients (6%).

Other reasons for discontinuations are: travel, adverse events, funeral, treatment with traditional remedies, or inability to pay for drugs. The reason for discontinuation was unknown for 657patients (79%).

Virologic failure: plasma HIV RNA>300 copies/ml.

*Overall p value.

**The sensitivity analysis uses an expanded definition of drug stock-out, which includes any treatment discontinuation that lasted more than one month, was not accompanied by an explanation in the medical record, occurred when the pharmacy reported a shortage of at least one of the drugs in the patient's cART regimen.

Results remained robust in the sensitivity analysis on the definition of drug stock-out. Stock-out-related discontinuations continued to be independently associated with interruption in care or death (adjusted HR, 3.09; 95% CI, 1.36–7.05) when we assumed that all treatment prolonged discontinuations during drug shortage periods, as reported by the pharmacy, were due to drug stock-outs, regardless of the explanation in the patient's medical record (**Table 3**).

When we restricted the analysis to only those patients whose initial cART regimen included drugs that had been out of stock (n = 405), discontinuations caused by drug stock-outs were independently associated with a higher risk of interruption in care or death compared to no discontinuations or modifications (adjusted HR, 5.23; 95% CI, 2.06–13.30), but stock-out-related regimen modifications were not (adjusted HR, 1.48; 95% CI, 0.47–4.61; **Table 4**).

**Table 4:** Factors independently associated with interruption in care or death among 405 HIV-infected patients whose initial cART regimen was affected by drug stock-outs, Abidjan, Côte d'Ivoire.

	Basecase Analysis					Sensitivity analysis**		
	Interruption in care/death, n (%)		HR	95% CI	p	HR	95% CI	p
	Yes	No						
**cART discontinuation or modification**								
None	14 (10.14)	124 (89.86)	1.00		**0.01** *	1.00		**<0.01** *
Modification/stock-out	4 (9.30)	39 (90.70)	1. 48	0.47–4.61	0.50	1. 49	0.48–4.66	0.50
Modification/other reasons	2 (10.53)	17 (89.47)	2.62	0.59–11.75	0.21	2.64	0.59–11.84	0.20
Discontinuation /stock-out	8 (17.78)	37 (82.22)	5.23	2.06–13.30	**<0.01**	5.54	2.17–14.15	**<0.01**
Discontinuation/other reasons	32(20.25)	126 (79.75)	2.89	1.50–5.57	**<0.01**	2.89	1.50–5.58	**<0.01**
**Gender**								
Female	38 (13.01)	254 (86.99)	1.00		**0.01**	1.00		**0.01**
Male	22 (19.82)	89 (80.18)	1.98	1.15–3.40		2.00	1.16–3.44	
**Study center**								
CePReF	18 (9.23)	177 (90.77)	1.00		0.10*	1.00		0.10*
CNTS	4 (25.00)	12(75.00)	1.97	0.62–6.32	0.25	1.99	0.62–6.40	0.25
Yopougon-Attie	38 (19.79)	154 (80.21)	1.97	1.05–3.71	**0.04**	2.01	1.06–3.80	**0.03**
**First-line cART regimen**								
ZDV/3TC/EFV	29 (11.65)	220 (88.35)	1.00		**<0.01** *	1.00		**0.01** *
d4T/3TC/EFV	14 (15.05)	79 (84.95)	1.86	0.95–3.65	0.07	1.87	0.95–3.69	0.07
ZDV/3TC/NVP	4 (23.53)	13 (76.47)	1.79	0.60–5.33	0.29	1.79	0.60–5.32	0.30
Other	13 (29.55)	31 (70.45)	3.22	1.63–6.36	**<0.01**	3.24	1.64–6.40	**<0.01**
**Virological failure (6 months)**								
No	38 (12.06)	277 (87.94)	1.00		**0.01**	1.00		**0.01**
Yes	22 (25.00)	66 (75.00)	2.05	1.16–3.60		2.04	1.16–3.57	

HR: hazard ratio, adjusted for all variables in the table;

CNTS: HIV clinic affiliated with the National Center for Blood Transfusion;

ZDV: zidovudine; 3TC: lamivudine; d4T: stavudine; EFV: efavirenz; NVP: nevirapine.

Other cART regimens: zidovudine-lamivudine-indinavir/ritonavir, lamivudine-stavudine-indinavir/ritonavir, or stavudine-abacavir-efavirenz.

Other reasons for modifications are: adverse events, tuberculosis, pregnancy and treatment failure. The reason for modification was unknown for 18 patients (6%).

Other reasons for discontinuations are: travel, adverse events, funeral, treatment with traditional remedies, or inability to pay for drugs. The reason for discontinuation was unknown for 657patients (79%).

Virologic failure: plasma HIV RNA>300 copies/ml.

*Overall p value.

**The sensitivity analysis uses an expanded definition of drug stock-out, which includes any treatment discontinuation that lasted more than one month, was not accompanied by an explanation in the medical record, occurred when the pharmacy reported a shortage of at least one of the drugs in the patient's cART regimen.

We evaluated the separate impacts of drug stock-outs on retention in care and deaths. We found an association between drug stock-outs and retention to care, but not between drug stock-outs and death (data not shown). The lack of association between drug stock-outs and death is likely due to a lack of statistical power.

## Discussion

In this study conducted in Abidjan, Côte d'Ivoire, we found that at least 11% of the 1,554 patients who started cART between February 1, 2006 and June 1, 2007 were affected by stock-outs of at least one of their antiretroviral drugs. Because we could no assess the reason for treatment discontinuation for a majority of patients, the proportion of those affected by stock-outs may be higher than 11%. Nevirapine and fixed-dose combination zidovudine/lamivudine were out of stock most frequently. Generic fixed-dose stavudine/lamivudine/nevirapine was always in stock. Prolonged discontinuations related to drug stock-outs were independently associated with a higher risk of interruption in care or death compared to no discontinuations or modifications. In contrast, stock-out-related regimen modifications did not have a significant impact on interruption in care or death.

Few studies have reported on the frequency and management of antiretroviral drug stock-outs in sub-Saharan Africa. In Côte d'Ivoire [6], [7] and Rwanda [5], studies focusing mainly on cART adherence reported that drug stock-outs led to 10%–28% and 52% of cART discontinuations. The large difference between the results of these studies may be related to the definition of drug stock-out used. Drug stock-outs may also change over time and by setting.

Drug stock-outs in this study can in part be explained by structural problems that affected all Aconda centres. The drug stock-outs that occurred at the time of the study were related to structural problems affecting all 20 Aconda centres. From May 2004 to January 2006, the HIV care centres included in the study received their drugs directly from the United States, through HEART PEPFAR. Starting in January 2006, the National HIV Care Program of Côte d'Ivoire established the Central Public Health Pharmacy (CPHP) as the main drug supplier for HIV care centres in Abidjan. The drug needs of all Aconda centres were thus sent to the CPHP, leading to antiretroviral drug supply interruptions between 2006 and 2007, due to the large volume of orders. Furthermore, PEPFAR did not consistently provide a sufficient amount of drugs to the CPHP, due to financial limitations. The current global economic crisis may lead government and international organizations to withdraw some of their funding from treatment programs in developing countries. As a result, drug stock outs and treatment interruptions may increase.

In this study, generic fixed-dose stavudine/lamivudine/nevirapine, which is often used for first-line cART in sub-Saharan Africa, was always in stock. We acknowledge that our results cannot be necessarily extrapolated to other settings and that stock-outs are likely to continue even with the establishment of a standard fixed-dose first line regimen. Nevertheless, in low-income countries, where access to cART is increasing rapidly, stock management may be facilitated and stock-outs may decline if national guidelines recommend one combination for all patients initiating first-line therapy. Harries *et al.* recently described a model of rational antiretroviral drug forecasting in Malawi [24]. This model is similar to the model adopted by the country's Tuberculosis Control Program, and suggests using fixed-dose stavudine/lamivudine/nevirapine as a standard first-line cART regimen. The authors did not report any antiretroviral drug stock-outs in Malawi during the study period, from July to December 2006. The WHO recently updated guidelines on antiretroviral therapy for HIV infection in adults and adolescents [25] recommend that countries in sub-Saharan Africa should develop a plan to move towards zidovudine or tenofovir-based first-line regimens rather than stavudine-based regimen while the goal of this is to avoid drug toxicity. The results of the current study suggest that first-line regimens should be standardized in this transition to decrease the probability of stock-outs.

During the study period, 12% of patients died and 23% interrupted care. Most of these events occurred in the first six months of cART. These estimates are consistent with those reported in other pilot cART programs [12], [14], [17], [18], [20], [26]. We evaluated the impact of stock-outs on interruption in care or death in the 975 patients who were on cART for at least six months. The risk of interruption in care or death was three times higher for patients who discontinued cART because of drug stock-outs, but stock-out-related cART modifications did not affect patient outcomes. Interventions to decrease the frequency of drug stock-outs, such as standard first-line cART regimens, should be implemented. Written protocols should recommend cART specific regimens in situations where first- and second-line cART regimens are out of stock. HIV centers should also keep alternative antiretroviral drugs in stock so that clinicians can modify regimens, rather than interrupting them, during stock-outs. In our study, physicians managed most stock-outs by modifying rather than interrupting therapy. Over the course of the study, when stock-outs occurred, the proportion of patients who discontinued treatment decreased and the proportion of patients who modified their regimens increased. This trend may illustrate physicians' tendency to capitalise on their experience in order to better manage drug stock-outs.

This study suggests that men are at higher risk of interruption in care and death than women, as previously reported [12], [17], [27], [28], [29]. Moreover, the rate of virologic response six months after treatment initiation, a well known predictor of efficacy and adherence [9], [30], [31], was independently associated with interruption in care or death. In 2008, De Beaudrap et al. reported in a Senegalese cohort, that body mass index and CD4 count were predictive of death during the first 6 months of follow-up after cART initiation, but after 6 months, the viral load was a stronger predictor of mortality and the effect of CD4 count was less pronounced [32].

Finally, as shown in previous studies, treatment modifications that were not related to drug stock-outs were independently associated with death [10], [12], [33]. Treatment discontinuations not due to stock-outs, but rather to non-adherence or other reasons, were also associated with interruption in care or death, as previously described [12], [16], [17], [34]. Cesar et al. recently reported in that almost 28% of 5,026 patients who initiated cART in a Caribbean and a Latin American cohort modified their first-line regimen one year after initiation, mainly because of adverse events [35]. Other studies conducted in resource-limited settings, have reported that 20–28% of patients modify their first-line regimen [36], [37]. In our study, 280 (28%) of 1,554 patients modified their cART regimens at least once during the follow-up (median 13 months). This result is consistent with data reported in the literature.

Of note, we also found an increased risk of interruption in care or death when patients initiated cART with abacavir or indinavir. Although data are limited, indinavir-containing regimens have been shown to be associated with lower rates of adherence than stavudine/lamivudine/nevirapine [38].

This analysis has several limitations. First, we collected stock-out data was retrospectively. We were not able, to assess the reason for treatment discontinuations for a majority of patients. We may, therefore, have underestimated the proportion of patients for whom treatment discontinuations were due to drug stock-outs. This limitation may have affected the association we found between stock-out-related discontinuations and interruption of care or death. We attempted to minimise bias by choosing the group of patients who did not interrupt cART as the reference. Furthermore, the association between stock-out-related cART discontinuations and interruptions of care or death remained robust when we modified the definition of drug stock-out (Table 3). Second, inclusion in the analysis of all patients on cART rather than just those taking antiretroviral drugs that were not always in stock may have attenuated the estimated association between drug stock-outs and interruption in care or death. When we only included patients on cART regimens affected by drug stock-outs, the risk of interruption in care or death was higher for patients who interrupted cART because of drug stock-outs, but not for patients who interrupted cART for other reasons. In this sensitivity analysis, stock-out-related regimen modifications were not associated with interruption in care or death.

In conclusion, this study suggests that drug stock-outs affect a substantial proportion of patients on cART in Abidjan, even though generic the fixed-dose recommended as first-line therapy in Côte d'Ivoire, stavudine/lamivudine/nevirapine, was always in stock. Stock-out-related cART discontinuations had a significant impact on retention in care and death, but cART regimen modifications did not. With the movement towards newer, less toxic first-line regimens in sub-Saharan Africa, it is crucial that those regimens be standardised to avoid drug stock-outs and their consequences.
